# Characteristics and comparison between e-scooters and bicycle-related trauma: a multicentre cross-sectional analysis of data from a road collision registry

**DOI:** 10.1186/s12873-022-00719-0

**Published:** 2022-09-29

**Authors:** Axel Benhamed, Amaury Gossiome, Amina Ndiaye, Karim Tazarourte

**Affiliations:** 1 Service SAMU-Urgences, Hospices Civils de Lyon, Centre Hospitalier Universitaire Édouard Herriot, 5 place d’Arsonval, 69003 Lyon, France; 2 INSERM U1290 (RESHAPE), Université Claude Bernard Lyon 1, 69003 Lyon, France; 3 IFSTTAR, Université Gustave Eiffel, 69675 Lyon, France

**Keywords:** Trauma, E-scooter, Bicycle, Road traffic accident

## Abstract

**Background:**

Urban mobility has drastically evolved over the last decade and micromobility rapidly became an expanding segment of contemporary daily transportation routines. E-scooter riders and bicyclists may share similar trauma characteristics, but this has been little explored. The objective was to describe and compare the characteristics of e-scooter and bicycle-related trauma.

**Methods:**

We conducted a cross-sectional analysis of data from the Rhône road collision registry (January 1, 2019 to December 31, 2019). We included all e-scooter or bicycle riders injured in traffic collisions during the study period; there were no exclusion criterion.

**Results:**

A total of 2,779 patients were included; 825 (29.7%) were e-scooter riders and 1,954 (70.3%) were bicyclists. E-scooter riders were younger (median [IQR]: 24 [20–32] vs 29 [20–45] years, *p* < 0.001) and less frequently male (64.2% vs 73.4%, *p* < 0.001). Most e-scooter and bicycle road collisions were consequent to a fall or loss of vehicle control (74.2% vs 67.7%, *p* < 0.001). E-scooter riders were less frequently wearing a helmet at the time of the road collision (6.1% vs 30.7%, *p* < 0.001) and had more frequently head (24.2% vs 19.9%, *p* = 0.01) and face (30.6 vs 20.5%, *p* < 0.001) injuries compared to bicyclists. The median injury severity score was 2 [1–4] in both groups with no significant difference (*p* = 0.77).

**Conclusions:**

E-scooter and bicycle-related trauma patients were mainly young males with minor injuries and most of them sustained a road collision with no third-party. However, they suffered from different injury patterns; e-scooter riders suffered more frequently face and head injuries than bicycle riders, which may be at least partly the consequence of less frequent helmet use among e-scooter riders compared to bicyclists. Hence the two groups of users should not be considered as a single trauma entity. This issue should be promptly addressed to bring down the incidence of preventable injuries and avoid healthcare costs.

**Supplementary Information:**

The online version contains supplementary material available at 10.1186/s12873-022-00719-0.

## Background

Urban mobility has drastically evolved over the last decade and micromobility rapidly became an expanding segment of contemporary daily transportation routines. A collective awareness of environmental issues combined with metropolitan traffic congestion have, among other factors, boosted its fast development [1, 2]. Electric-powered two-wheeled devices are increasingly popular and the recent expansion of electric scooter (also called “e-scooters”) sharing companies in major cities across the world has led to a substantial increase in the use of such vehicles, both for daily transportation and recreational rides [3, 4]. Furthermore, it is an easy-to-use and eco-friendly means of transportation [5] that offers fast travel (up to 25 km/h) and the possibility to avoid traffic jams thanks to its compact size [6] as is also possible using a bicycle. It has therefore become a popular low-cost alternative to public transportation for short trips, and this has been recently enhanced by the COVID-19 pandemic-related social distancing rules [7]. Nevertheless, this hybrid vehicle combines the characteristics of two-wheel human-powered vehicles (such as a bicycle) with those of motorized ones (such as a motorcycle). French legislation considers e-scooters as bicycles as long as they do not exceed 25 km/h and users of such e-scooters are allowed to ride on the sidewalk as well as on the road (as long as speed limit does not exceed 80 km/h) [8]. However, the characteristics of e-scooter road collisions do not fully match with features of bicycle road collisions in terms of crash typologies, demographics, and spatial and temporal distribution [9]. It is therefore unclear whether clinicians could appropriately consider these two groups of vehicles as a single entity in terms of trauma characteristics. Indeed, e-scooters are in a relative unique position where they are sufficiently compact to negotiate pedestrian traffic on sidewalks (while it is forbidden for bicyclists), yet fast enough to ride on roadways. Hence, e-scooters are a very controversial means of transportation and are considered as potentially unsafe because of poor visibility and lack of dedicated lanes. Another major source of concern is that no driving license is needed for their use or rental in many countries, but also riders are not consistently required to wear a helmet and when they are (according to local legislation) they do so infrequently [10]. In addition, riders may also engage in inappropriate behaviour such as driving under the influence of alcohol/drugs or double-riding on the same vehicle [6]. As a consequence, the number of patients admitted to an emergency department (ED) after a e-scooter accident-related trauma has dramatically increased in developed countries [11, 12] leading to significant healthcare costs [13]. Head and limb injuries have been widely reported as the two most common injured body regions [10, 14]. Yet, e-scooter trauma characteristics, especially in Europe [14], have been poorly described and only one study compared e-scooter to non-motorized bicycle road collisions characteristics [15]. We therefore conducted a study to describe and compare the characteristics of e-scooter and bicycle-related trauma.

## Methods

### Study design

We conducted a cross-sectional analysis of data from the Rhône Road collision registry between January 1, 2019, and December 31, 2019.

### Setting

The Rhône road collision registry (*Registre des victimes d'accidents de la circulation du Rhône*) prospectively records data from implemented in 1995 and covers the Rhône area of France (1.85 million inhabitants) including one of the largest cities in France (Lyon, 0.5 million inhabitants, 11,000 inhabitants/km^2^). Patients are included in the registry if they sustained a road traffic injury involving one or more vehicle (motorized or not) in the Rhône area and required institutional healthcare from one of the 245 private or public healthcare structures (including 42 ED and 20 intensive care units [ICU] within level-I, -II and -III trauma centres) cooperating together, including prehospital primary care teams and forensic medicine institutes.

The registry collects the demographic characteristics of each road collision casualty and a description of the sustained body injuries. Patient information is collected prospectively from the accident site to hospital discharge: prehospital emergency care at the scene, ED, ICU, surgery units, and discharge including. The full data collection method has been described elsewhere [16]. Results are reported as per the STROBE guidelines (Supplementary Table) [17].

### Participants

We included all e-scooter or bicycle riders injured in traffic collisions during the study period; there were no exclusion criterion.

### Variables

We extracted and analysed the following variables: age, sex, road rider category (e-scooter or bicycle), trauma characteristics including antagonist, time of day, season, safety equipment (helmet), anatomical injuries by body region based on the Abbreviated Injury Scale (AIS), Injury Severity Score (ISS), and outcome (ICU admission and in-hospital mortality). The outcomes were epidemiological characteristics, injury pattern, and in-hospital mortality.

### Data sources/measurement

Each injury is coded according to the AIS (2005 update version), a severity score that ranges from one (minor) to six (beyond treatment). As a given patient can have suffered multiple injuries in the same body region we used the maximum AIS (MAIS) that is the severity score of the subject’s most severe injury in each body region. The ISS is calculated from the three worst-affected body regions as the sum of squares of the respective AIS.

### Statistical methods

We performed descriptive analyses. Baseline characteristics were described by frequencies and percentages for categorical variables, and medians and interquartile range [IQR] for continuous variables. We compared the two groups using the Pearson Chi^2^ test for categorical variables and the Student-t test for continuous variables. Missing data were not imputed. In all analyses, *p* < 0.05 was considered as significant. Statistical analyses were performed using SAS (Statistical Analysis System v9.4, SAS Institute Inc., Cary, NC, USA).

## Results

### Patient characteristics

A total of 2,779 patients were included; 825 (29.7%) were e-scooter riders and 1,954 (70.3%) were bicyclists. E-scooter riders were younger (24 [20–32] *vs* 29 [20–45] years old, *p* < 0.001) and less frequently male (64.2% *vs* 73.4%, *p* < 0.001). Patients aged 45–65 years constituted 8.9% of e-scooter riders and those aged 65 years or over 0.5%; these proportions were higher among bicyclists (20.1% and 5.3% respectively, *p* < 0.001). Most road collisions involving an e-scooter were consequent to a fall or loss of vehicle control and did not involve any other third party (69.7%) but this proportion was higher among e-scooter riders (74.2% *vs* 67.7%, *p* < 0.001). A car was involved less frequently in e-scooter-related collisions (13.9%) than bicycle-related collisions (17.1%; *p* = 0.036). In both groups the accident mostly occurred on a weekday (66.3% *vs* 69.0%, *p* = 0.035). Fewer accidents occurred during the winter season in the e-scooter group (7.9% *vs* 17.8%, *p* < 0.001). Most road collisions occurred on city streets, and this was more frequently the case for e-scooter users (98.8%) than bicyclists (91.5%, *p* < 0.001). Helmet use at the time of the collision was less frequent among e-scooter riders (6.1%) than among bicyclists (30.7%, *p* < 0.001; Table 1).

**Table 1:** Patient and road collision characteristics according to type of user

	E-scooter, *n* = 825 (29.7)	Bicycle, *n* = 1,954 (70.3)	*p*	Total population *n* = 2,779
Age, years, median [IQR]	24 [20–32]	29 [20–45]	**< 0.001**	27 [20–41]
0–9	14 (1.7)	128 (6.6)	**< 0.001**	142 (5.1)
10–29	551 (66.8)	881 (45.1)	**< 0.001**	1,432 (51.5)
30–44	183 (22.2)	448 (22.9)	0.67	631 (22.7)
45–64	73 (8.9)	393 (20.1)	**< 0.001**	466 (16.8)
≥ 65	4 (0.5)	104 (5.3)	**< 0.001**	108 (3.9)
Sex, male	534 (64.2)	1435 (73.4)	**< 0.001**	1,969 (70.9)
Third party				
None	612 (74.2)	1,324 (67.7)	**< 0.001**	1,936 (69.7)
Stationary item^a^	74 (9.0)	169 (8.7)	0.78	243 (8.7)
Bicycle, scooter, pedestrian, skateboard	13 (1.6)	74 (3.8)	**0.002**	87 (3.1)
Motorcycle	7 (0.9)	14 (0.7)	0.71	21 (0.8)
Car	115 (13.9)	335 (17.1)	**0.036**	450 (16.2)
Bus, train, truck, tram	4 (0.5)	31 (1.6)	**0.018**	35 (1.3)
Time of day, *n* = 1,610				
Morning, 6AM-11.59AM	76 (20.0)	85 (7.0)	**< 0.001**	395 (24.6)
Afternoon, 12AM-5.59PM	123 (32.4)	319 (26.1)	0.35	620 (38.5)
Evening, 6PM-11.59PM	101 (26.6)	497 (40.8)	**< 0.001**	419 (26)
Night, 12AM-5.59AM	91 (24.0)	319 (26.1)	**< 0.001**	176 (10.9)
Day of the week, *n* = 2,763				
Weekdays	534 (66.3)	1,345 (69.0)	**0.035**	1,879 (68.0)
Weekend	279 (34.7)	605 (31.0)	0.14	884 (32.0)
Season, *n* = 2,763				
Spring	269 (33.1)	569 (29.2)	0.067	838 (30.3)
Summer	248 (30.5)	642 (32.9)	**< 0.001**	890 (32.2)
Fall	232 (28.5)	391 (20.1)	**< 0.001**	623 (22.6)
Winter	64 (7.9)	348 (17.8)	**< 0.001**	412 (14.9)
Type of road, *n* = 2,639				
City street	763 (98.8)	1,708 (91.5)	**< 0.001**	2,471 (93.6)
Secondary road	0 (0)	56 (3.0)	**-**	56 (2.1)
Primary road	0 (0)	2 (0.1)	**-**	2 (0.08)
Freeway	0 (0)	1 (0.05)	**-**	1 (0.04)
Other (country/forest path…)	9 (1.2)	100 (5.4)	**< 0.001**	109 (4.1)
Helmet, yes, *n* = 2,686	50 (6.1)	572 (30.7)	**< 0.001**	622 (23.2)

Bold *p* values denote a significant difference between groups

^a^Including parked vehicles

## Trauma characteristics and outcome

Upper extremities, lower extremities, face, and head were the most frequent body region injured in both groups; head (24.2% *vs* 19.9%, *p* = 0.01) and face (30.6% *vs* 20.5%, *p* < 0.001) injuries were more frequent among e-scooter riders compared to bicyclists. Conversely, upper limb injuries were less frequent (48.9% *vs* 57.6%, *p* < 0.001). Among patients with no helmet at the time of the collision, e-scooter riders sustained more frequently AIS ≥ 1 face/head injuries compared to bicycle riders (45.2% *vs* 35.3%, *p* < 0.001). As regards e-scooter riders who wore a helmet at the time of the collision, they presented with such injuries in a smaller proportion compared to those who did not (24.0% *vs* 45.2%, *p* = 0.003) while there was no difference among bicycle riders (31.6% *vs* 35.3%*, p* = 0.13*)*.

Less than 2% of patients had severe injuries (AIS ≥ 3) in each of the two groups and the only notable difference in the distribution according to body region was for the head (1.9% in the e-scooter group *vs* 1% in the bicycle group, *p* = 0.04). The second most frequent body region severely injured was the lower limbs (1.5% *vs* 1.7%, *p* = 0.66). No e-scooter rider sustained severe injuries to the face, the spine, or upper limbs. The median [IQR] ISS was 2 [1-4] in both groups (

There was no notable difference in the proportion of riders who underwent surgery (7.8%, *n* = 64 e-scooter riders *vs* 7.5% *n* = 146 bicyclists, *p* = 0.81). A total of 2.1% (*n* = 17) of the e-scooter riders and 1.7% (*n* = 34) bicyclists were admitted to an ICU (*p* = 0.27); respectively, 0.1% (*n* = 1) and 0.2% (*n* = 4) died (*p* = 1; Table 2).

**Table 2:** Trauma characteristics and outcome according to type of user

	E-scooter, *n* = 825 (29.7)	Bicycle, *n* = 1,954 (70.3)	*p*
Injury pattern (AIS ≥ 1^a^)			
Head	200 (24.2)	389 (19.9)	**0.01**
Face	252 (30.6)	401 (20.5)	**< 0.001**
Neck	27 (3.3)	49 (2.5)	0.26
Thorax	60 (7.3)	176 (9.0)	0.14
Abdomen/pelvis	28 (3.4)	63 (3.2)	0.82
Spine	55 (6.7)	154 (7.9)	0.27
Upper extremities	403 (48.9)	1,126 (57.6)	**< 0.001**
Lower extremities	345 (41.8)	758 (38.8)	0.14
External	35 (4.2)	125 (6.4)	**0.03**
Injury pattern (AIS ≥ 3^a^)			
Head	16 (1.9)	20 (1.0)	0.05
Face	0 (0)	2 (0.1)	-
Neck	1 (0.1)	2 (0.1)	0.56
Thorax	8 (1.0)	23 (1.2)	1
Abdomen/pelvis	2 (0.2)	8 (0.4)	-
Spine	0 (0)	2 (0.1)	-
Upper extremities	0 (0)	2 (0.1)	-
Lower extremities	12 (1.5)	33 (1.7)	0.66
External	0 (0)	0 (0)	-
Injury severity score, median [IQR]	2 [1–4]	2 [1–4]	0.77
Need for surgery	64 (7.8)	146 (7.5)	0.81
Intensive care unit admission	17 (2.1)	34 (1.7)	0.26
In-hospital mortality	1 (0.1)	4 (0.2)	1

Bold *p* values denote a significant difference between groups

A patient could have suffered from multiple injuries, therefore the total of injuries (*n* = 8,729) presented in the table is greater than the number of patients (*n* = 2,779)

^a^Abbreviated injury scale

## Discussion

### Principal findings

In the present study we found that e-scooter-related trauma patients were numerous in 2019 in the Rhône department. They accounted for nearly a third of combined e-scooter/bicycle-related ED admissions. They shared characteristics (no third party involved, mostly mild injuries to the extremities) and outcome (very few ICU admission and very low mortality rate) with bicyclists. However, e-scooter riders sustained more frequently face and head injuries compared to bicyclists, and, conversely, fewer injuries to the upper extremities. We also noted that less than one in ten e-scooter riders wore a helmet while five times more bicyclists wore one at the time of the road collision.

### Clinical interpretation and comparison with previous studies

Both e-scooter and bicycle riders were mostly male young adults and the proportion of older adults (≥ 65 years) among e-scooter riders was ten times lower than bicycle riders. This is consistent with that reported elsewhere [13, 18, 19] and reflects both the urban implementation of e-scooter sharing companies and their target customers’ profile. More interestingly, we found that a moving car was less frequently involved in e-scooter crashes compared to cycling incidents, which has also been reported by Cicchino *et al*. (13.1% vs. 37.7%) [15]; we assume that e-scooter riders drove more frequently on the sidewalk. Consistent with this, being hit by a moving vehicle or an object was reported in only 8.8% cases while fall was the most common mechanism (80.2%) in e-scooter crashes in the study reported by Trivedi *et al*. [18].

A recent scoping review [14] suggested that the extremities, face, and head were particularly vulnerable in e-scooter falls or collisions, while injuries to the chest (1–10%) and abdomen (0–6%) are less common. Herein, these body areas were also the least frequently injured (< 10% herein), and we also noted that they were primarily mild injuries. This is certainly related the absence of third-party involvement in most cases, especially motorized vehicles; it may also partially explains why few abdominal/thoracic injuries were reported and why the overall patient severity (based on the widely use ISS) was, as reported elsewhere [14], low. A recent review of the literature found that, although most head injuries were mild/concussions, approximately 15% involved intracranial haemorrhage or skull fractures [10]. Herein, we noted that e-scooter riders sustained twice more frequently a severe injury to the head compared to bicyclists. In addition, we noted a substantial proportion of patients presenting with a facial injury as it has been noted elsewhere [20]. These findings are probably related to infrequent use of a helmet by e-scooter riders herein, which was five times less than bicyclists. The scoping review cited above (16 studies; *n* = 1,656) also showed that only 4.5% of e-scooter riders wore a helmet [14] and the difference between the two groups of riders can be as high as 2% *vs* 66.4% (*p* < 0.001) [15]. Several factors could explain this. First, e-scooter patients were younger, and consequently we assume that fewer of them had a driver’s licence and therefore were educated on the highway code. In line with this hypothesis, it has been found that almost half of e-scooter trauma patients did not have a driver’s licence [19]. In addition, it is reported that e-scooter riders are more likely to be casual users renting/using the vehicle for social purposes (35.4% *vs* 10.7%) and less frequently regularly use these to commute to/from work (25.3% *vs* 52.1%) compared to bicyclists [15]. We also assume that regular users are more vigilant than occasional ones. However, helmets have been found to be effective in protecting against serious and fatal head injury, as well as other head injury and face injury [21]. Wearing a helmet is also associated with reduced mortality [21], which raises the question of making helmets mandatory for e-scooter riders. This is further supported by Hoye *et al*. who conducted a meta-analysis and found that mandatory bicycle helmet legislation for all cyclists was associated with a significant reduction (-20%, 95% CI [-27; -13]) in head injuries, and a larger effect was found for serious head injury (-55%, 95% CI [-78; -8]) [22]. It has also been reported that most frequent circumstantial factors associated with bicycle-related trauma were helmet and alcohol use [22]. Herein, toxicology data were not collected, but other authors reported that such dangerous behaviour was very frequent; 48% e-scooter crashes cases were associated positive alcohol screening and 52% with positive urine toxicology [23]. Bai *et al*. also assessed risky behaviour in micromobility riders and specifically reported that e-scooter riders were more likely to ride in motorized lanes and against traffic compared to bicyclists (e-bike or not) [24]. In accordance with Farley *et al*., we do believe that it is of the utmost importance to investigate different strategies, such as mandatory helmet, light and bell use, maximum velocity limitation, legislation enforcement, and reinforcement against riding under the influence of any toxic substance, to potentially mitigate the most serious injuries and keep riders safe [11].

The overall burden of care related to the introduction of e-scooters has been evaluated in a study conducted in New Zealand; over a period of seven months, a total of 770 road collisions led to 246 ED admissions, 569 hospital bed-hours, 441 inpatient scans, and 49 operations [13]. The authors clearly showed that e-scooters implementation had a significant impact both on the primary urban trauma centre as well as community care facilities, and they also estimated a mean cost per injury of $NZ 1,693 [13].

### Strengths and weaknesses of the study

A major strength of this study is that it is multicentre, based on prospectively collected data from urban and rural facility care including level-I to III trauma centres and patients of all ages. Another strength is that it is based on an exhaustive road trauma registry [25]. However, the study does have certain limitations. First, because of the design of the study, sample size calculation was not relevant. Furthermore, the results were not adjusted for multiple testing. For both reasons, the significant differences must be interpreted with caution and the impact of wearing a helmet could not be proven herein. Second, we were not able to provide a comprehensive picture of trauma characteristics; for instance, we did not collect information regarding the travel purpose, whether patients owned their own vehicle or were renting it, whether they were single or double riders, whether they were under the influence of alcohol/drugs, or whether the user was at fault in the accident. In addition, the registry does not record data pertaining to clinical evaluation (such as Glasgow coma scale score, physiological parameters) or medical management that could have influenced patient outcome. This study may also suffer from underreporting of road traffic injuries; patients had to consult or be addressed to an ED to be included in the registry, and therefore some patients may have not been captured. but we hypothesize that they sustained minor injuries.

### Implications for clinicians or policymakers

Our findings suggest that clinicians should always suspect thoracic, spinal, and abdominal injuries in patients sustaining e-scooter-related trauma. Although rare, these patients may also suffer from severe traumatic brain injuries and should not systematically be considered as minor trauma. In addition, this study could help policymakers to tighten legislation mandating the use of protective equipment such as a helmet, promote education program targeting inexperienced riders, and develop the construction of e-scooter and bicycle riders dedicated pathways, especially in urban areas.

### Unanswered questions and future research

Future research on micromobility-related trauma would benefit from an economic evaluation to measure e-scooter and bicycle-related trauma healthcare associate costs. In addition, prospective studies are needed to assess the impact of wearing a helmet among e-scooter riders. Measuring and characterising inappropriate behaviour (concurrent intoxication from alcohol and illicit drugs, double-riding …) could be of help to target future road safety policies.

## Conclusion

E-scooter and bicycle riders who sustained a trauma were mainly young males involved in road collisions with no third-party, and most of them had minor injuries. However, different injury patterns were found; e-scooter riders sustained more frequently face and head injuries, which may be at least partly the consequence of very low helmet use compared to bicyclists. Hence the two groups of users should not be considered as a single trauma entity. This issue should be promptly addressed to bring down the incidence of preventable injuries and avoid healthcare costs.

## Supplementary Information

**Additional file 1. **STROBE Statement—Checklist of items that should be included in reports of cross-sectional studies.

## Data Availability

The datasets which were analysed during the current study are available from the contributing author Amina Ndiaye on reasonable request at amina.ndiaye@univ-eiffel.fr.

## Abbreviations

AIS: Abbreviated injury scale
ED: Emergency department
ICU: Intensive care unit
IQR: Interquartile range
ISS: Injury severity score
MAIS: Maximum abbreviated injury scale
